# Meta-Analysis Results on the Association Between TP53 Codon 72 Polymorphism With the Susceptibility to Oral Cancer

**DOI:** 10.3389/fphys.2018.01014

**Published:** 2018-08-02

**Authors:** Ying-Mei Lin, Jun Shao, Xiao-Hong Yin, CaiCai Huang, Xiao-Wei Jia, Ya-Di Yuan, Chang-Jing Wu, En-Ming Zhen, Zhong-Xiong Yao, Xian-Tao Zeng, Rui-Hua Liu

**Affiliations:** ^1^Department of Stomatology, Guangzhou Hospital of Integrated Traditional and West Medicine, Guangzhou, China; ^2^Center for Evidence-based and Translational Medicine, Zhongnan Hospital of Wuhan University, Wuhan, China; ^3^Department of Gynecology, Guangdong Women and Children Hospital, Guangzhou, China

**Keywords:** TP53 codon 72, susceptibility, oral cancer, polymorphism, meta-analysis

## Abstract

**Objectives:** TP53 is an important tumor suppressor gene to maintain genomic integrity, and its mutations increase the susceptibility to oral carcinoma. Previous published studies have reported the relation of TP53 codon 72 polymorphism with the risk of oral carcinoma, but the results remain controversial and inconclusive.

**Methods:** We therefore utilized meta-analysis based on a comprehensive search in PubMed, EMBASE, and Google of Scholar databases up to August 19, 2017.

**Results:** Total 3,525 cases and 3,712 controls from 21 case-control studies were selected. We found no significant association between TP53 codon 72 polymorphism and oral carcinoma susceptibility in all genetic contrast models, including subgroup analysis based on control source and ethnicity. Furthermore, TP53 codon 72 polymorphism was not significant associated with oral carcinoma susceptibility in tobacco or alcohol use, and HPV infection status. Our results were confirmed by sensitivity analysis and no publication bias was found.

**Conclusions:** Taken together, our data indicate that TP53 codon 72 polymorphism is not associated with the susceptibility to oral carcinoma.

## Introduction

Based on the GLOBOCAN2012 investigations, oral carcinoma is regarded as one of the most common causes of cancer related morbidity and mortality, contributing to 3.8% of all cancer cases and 3.6% of cancer related deaths (Warnakulasuriya, 2009; Ferlay et al., 2015; Shield et al., 2017). The long-term survival rate of oral carcinoma is < 50% despite improved treatment schedules such as surgery, radiation and chemotherapy (Coleman et al., 2008; De Angelis et al., 2014). Oral carcinoma is highly associated with tobacco smoking, alcohol consumption and the exposure to a variety of exogenous or endogenous carcinogens (Petti, 2009). However, the etiology of oral carcinoma remains poorly understood. Furthermore, not all individuals exposing to these risk factors are subject to oral carcinoma, and additional genetic factors may also contribute to oral carcinoma susceptibility (Chen et al., 2010; Anantharaman et al., 2011; Niu et al., 2012).

The human TP53 is well-known tumor suppressor gene and plays an important role in DNA damage response by inducing cell cycle arrest or apoptosis (Slee et al., 2004; Harris and Levine, 2005). TP53 mutation is frequently found in human tumors, including oral carcinoma (Olivier et al., 2002). A common single nucleotide polymorphism at TP53 codon 72 is crucial for its tumor suppressor function (Suzuki and Matsubara, 2011). Several meta-analysis demonstrated that TP53 codon 72 polymorphism was associated with the susceptibility to a variety of cancers, such as colorectal cancer (Du et al., 2017), esophageal cancer (Steccanella et al., 2017), nasopharyngeal cancer (Zhuo et al., 2009a), and non-Hodgkin lymphomas (Xu et al., 2017).

Själander et al. demonstrated that the distribution of TP53 genotypes differed among different ethnicities, which is a notable confounding factor in carcinoma risk (Själander et al., 1995). Tobacco and alcohol use are known risk factors for oral carcinoma (Hashibe et al., 2007). In addition, TP53 codon 72 mutation spectrum has been shown to be altered with Human papillomavirus (HPV) infection, an emerging oral carcinoma risk factor (Chor et al., 2016). So far many case-control studies investigated the association of functional polymorphism of TP53 codon 72 with susceptibility to oral carcinoma, but the results remain conflicting and inconclusive (Tandle et al., 2001; Nagpal et al., 2002; Hsieh et al., 2005; Wang et al., 2012; Saleem et al., 2013). We therefore conducted this meta-analyses to evaluate the relationship of TP53 codon 72 polymorphism with tobacco and/or alcohol use and HPV infection in the susceptibility to oral carcinoma.

## Materials and methods

### Search strategy

PubMed, EMBASE, and Google of Scholar databases up to August 19, 2017 were searched with a combination of the keywords as follows: [(oral OR tongue OR mouth OR buccal OR oropharynx) AND (tumor OR carcinoma OR cancer) AND (TP53 OR P53 OR Arg72Pro) AND (variant^*^ OR mutation OR polymorphism^*^)].

### Inclusion and exclusion criteria

Inclusion criteria were: (i) evaluated the association between tobacco and/or alcohol uses, TP53 codon 72 polymorphism, HPV infection, and susceptibility to oral carcinoma; (ii) case-control researches published in English or Chinese; (iii) definite histopathologic diagnosis or clearly reported the type; (iv) sufficient data to evaluate the ORs and 95%CI, and *P*-value; (v) genotype distribution was in Hardy-Weinberg equilibrium (HWE). Major exclusion criteria were: (i) Reviews, conference abstracts, case reports; (ii) only-case study; (iii) genotype distribution was inconsistent with HWE; (iv) when duplicated studies published, only the study with the large sample size was included.

### Data extraction

Data extraction was performed independently by two authors using a standardized form. Data such as: first author, country, ethnicity, year of publication, source of the controls, genotype distribution of cases and controls. Discrepancies were settled by discussion, with disagreements resolved by consensus.

### Statistical analysis

The association was determined by calculating odds ratios (ORs) with corresponding 95% credible interval (95%CI). *Q*-test and *I*^2^ statistics were used to quantify statistical heterogeneity. The random-effect model was conducted if the heterogeneity was significant (*P* < 0.05) (DerSimonian and Laird, 1986); otherwise, the fixed effect model was utilized (Mantel and Haenszel, 1959). The sensitivity analysis was carried out through sequential exclusion of any one individual study. Begg's funnel plot and the Egger's test was performed to assess the potential publication bias of the researches (Begg and Mazumdar, 1994; Egger et al., 1997). The present meta-analysis was carried out by STATA 12.0 (Stata, College Station, TX, USA). *P* < 0.05 was considered statistically significant.

## Results

### Characteristics of the selected studies

**Figure 1:**
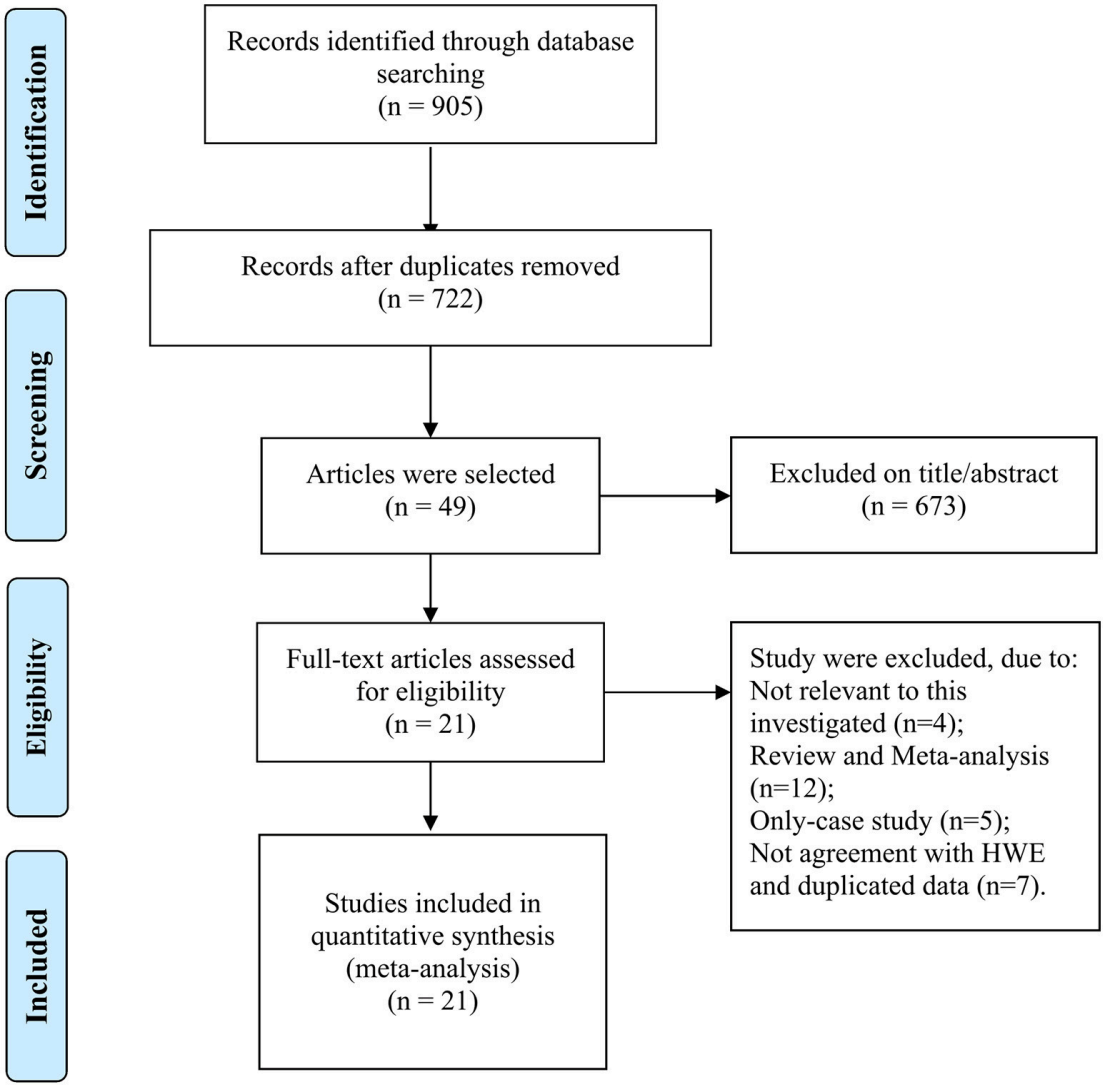
Flow diagram of the publication selection process.

The selection of eligible studies to be included in this meta-analysis was shown in Figure 1, 905 potentially relevant researches were initially obtained from the PubMed, EBMASE, and Google of Scholar databases. After the exclusion of irrelevant studies, a total of 28 studies were included. Among the remaining articles, four articles (Tandle et al., 2001; Jing et al., 2012; Saleem et al., 2013; Nagam et al., 2017) were not in agreement with HWE (*P* < 0.001) and three duplicated data publications were further excluded (Ji et al., 2008; Misra et al., 2009; Wang et al., 2012). We included 21 case-control study involving 3,525 oral carcinoma patients and 3,712 controls (Summersgill et al., 2000; Drummond et al., 2002; Nagpal et al., 2002; Shen et al., 2002; Katiyar et al., 2003; Kietthubthew et al., 2003; Hsieh et al., 2005; Mitra et al., 2005; Bau et al., 2007; Kuroda et al., 2007; Chen et al., 2008; Lin et al., 2008; Tu et al., 2008; Kitkumthorn et al., 2010; Ihsan et al., 2011; Saini et al., 2011; Patel et al., 2013; Adduri et al., 2014; Sina et al., 2014; Rao et al., 2017; Zarate et al., 2017). The characteristics of the selected studies are shown in Table 1.

**Table 1:** Main characteristics of studies included in the meta-analysis.

**Author**	**Year**	**Country**	**Ethnicity**	**Control source**	**Genotyping methods**	**Case**			**Control**			**HWE**
						**AA**	**AB**	**BB**	**AA**	**AB**	**BB**	
Adduri	2014	India	Asian	HP	PCR	23	48	44	31	53	26	0.7185
Patel	2013	India	Asian	HP	PCR-RFLP	32	29	18	30	58	22	0.5281
Kietthubthew	2003	Thailand	Asian	PB	PCR	32	44	21	35	34	28	0.0036
Nagpal	2002	India	Asian	PB	PCR	31	58	21	13	11	2	0.0876
Mitra	2005	India	Asian	HP	PCR	87	155	66	85	159	98	0.2031
Sina	2014	Iran	Asian	HP	PCR	20	25	10	40	48	12	0.6769
Ihsan	2011	India	Asian	PB	PCR	30	63	23	63	143	72	0.6186
Chen	2008	USA	Caucasian	PB	PCR-RFLP	183	121	22	181	144	24	0.5182
Zarate	2017	Argentine	Caucasian	HP	PCR	12	23	9	13	3	2	0.0471
Bau	2007	China	Asian	HP	PCR	46	70	21	18	65	22	0.014
Katiyar	2003	India	Asian	HP	PCR	10	24	10	5	12	3	0.3428
Saini	2010	Malaysia	Asian	PB	PCR	22	40	37	28	39	23	0.2152
Rao	2017	India	Asian	PB	PCR	35	110	59	46	112	54	0.1814
Lin	2008	China	Asian	PB	PCR	96	155	46	72	152	56	0.1352
Kuroda	2007	Japan	Asian	HP	PCR-RFLP	41	44	15	109	117	45	0.1591
Summersgill	2000	USA	Mixed	HP	PCR-CTPP	102	70	18	168	112	28	0.1436
Shen-a	2002	USA	Caucasian	PB	PCR-RFLP	55	41	9	175	134	24	0.8107
Shen-b	2002	USA	Caucasian	PB	PCR-RFLP	66	47	8	175	134	24	0.8107
Tu	2008	China	Asian	PB	DNAsequence	53	106	30	41	60	15	0.3367
Drummond	2002	Brazil	Mixed	NR	PCR	31	45	6	33	45	4	0.0212
Kitkumthorn	2010	Thailand	Asian	PB	PCR-RFLP	35	40	3	27	47	20	0.9569
Hsieh-a	2005	China	Asian	PB	PCR-RFLP	149	274	100	128	177	66	0.7229
Hsieh-b	2005	China	Asian	PB	PCR-RFLP	38	54	14	128	177	66	0.7229

*PB, population-based; HB, hospital-based; HWE, Hardy-Weinberg equilibrium; NR, no report*.

### Meta-analysis results

**Figure 2:**
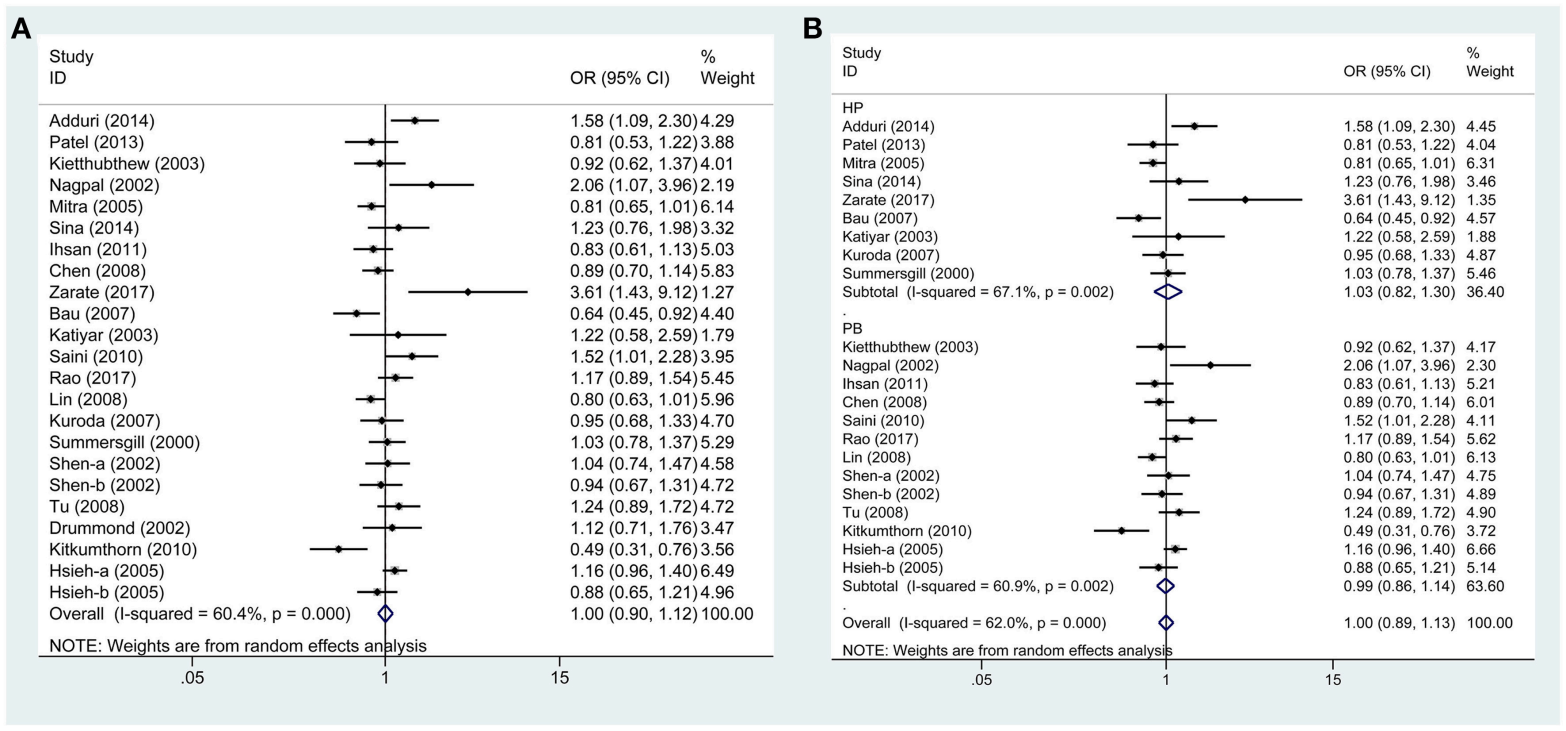
Forest plots demonstrated the association between TP53 codon 72 polymorphism and oral carcinoma susceptibility in the allele model. **(A)** Overall analysis. **(B)** Subgroup analysis by source of control.

Based on 21 case-control studies no significant association was found between TP53 Arg72Pro polymorphism and susceptibility to oral carcinoma in any genetic model (ArgPro vs. ArgArg: OR = 1.0, 95%CI = 0.90–1.11; ProPro vs. ArgArg: OR = 0.97, 95%CI = 0.84–1.12; Pro vs. Arg: OR = 1.0, 95%CI = 0.90–1.12; ArgPro+ProPro vs. ArgArg: OR = 1.01, 95%CI = 0.86–1.18; ProPro vs. ArgPro+ArgArg: OR = 0.96, 95% = 0.85–1.09). Based on subgroup analysis, stratified by control source or ethnicity, we obtained similar results (Figure 2, Table 2).

**Table 2:** Meta-analysis of the association between TP53 codon 72 polymorphism and oral carcinoma susceptibility.

**Comparison**	**Subgroup**	**Studies**	**Heterogeneity test**		**Association test**		**Model**	**Publication bias**
			***P*-value**	***I*^2^(%)**	**OR (95%CI)**	***P-*value**		**Egger**
Pro vs. Arg	Overall	21	0	60.4	1.00 (0.90–1.12)	0.953	R	0.16
	PB	13	0.002	60.9	0.99 (0.86–1.14)	0.898	R	
	HP	9	0.002	67.1	1.03 (0.82–1.30)	0.779	R	
	Caucasian	4	0.039	64.3	1.07 (0.78–1.47)	0.662	R	
	Asian	17	0	65.8	0.99 (0.86–1.14)	0.867	R	
ArgPro vs. ArgArg	Overall	21	0.03	39	1.00 (0.90–1.11)	0.991	F	0.355
	PB	13	0.241	20.1	1.04 (0.90–1.21)	0.59	R	
	HB	9	0.011	59.8	0.93 (0.67–1.29)	0.661	R	
	Caucasian	4	0.024	68.3	1.08 (0.69–1.69)	0.742	R	
	Asian	17	0.052	38.9	0.98 (0.84–1.19)	0.999	R	
ProPro vs. ArgArg	Overall	21	0.001	54.4	0.97 (0.84–1.12)	0.997	R	0.399
	PB	13	0.005	57.8	0.98 (0.73–1.32)	0.889	R	
	HB	9	0.015	57.8	1.02 (0.68–1.55)	0.913	R	
	Caucasian	4	0.317	15	1.09 (0.68–1.73)	0.722	R	
	Asian	17	0	63.5	0.96 (0.72–1.28)	0.775	R	
ArgPro+ProPro vs. ArgArg	Overall	21	0.001	53.8	1.01 (0.86–1.18)	0.914	R	0.266
	PB	13	0.028	47.7	1.03 (0.86–1.23)	0.752	R	
	HB	9	0.002	66.4	0.98 (0.70–1.37)	0.913	R	
	Caucasian	4	0.013	72.2	1.12 (0.71–1.77)	0.611	R	
	Asian	17	0.002	56.2	0.99 (0.82–1.20)	0.961	R	
ProPro vs. ArgArg+ArgPro	Overall	21	0.033	38.4	0.96 (0.85–1.09)	0.521	F	0.356
	PB	13	0.046	43.6	0.94 (0.81–1.10)	0.461	F	
	HB	9	0.084	42.6	0.98 (0.79–1.21)	0.846	F	
	Caucasian	4	0.82	0	1.06 (0.71–1.59)	0.77	R	
	Asian	17	0.005	52.9	0.97 (0.78–1.20)	0.761	R	

*OR, odds ratio; CI, confidence interval; F, fixed-effects model; R, random-effects model; NA, not available; PB, population-based; HB, hospital-based*.

**Figure 3:**
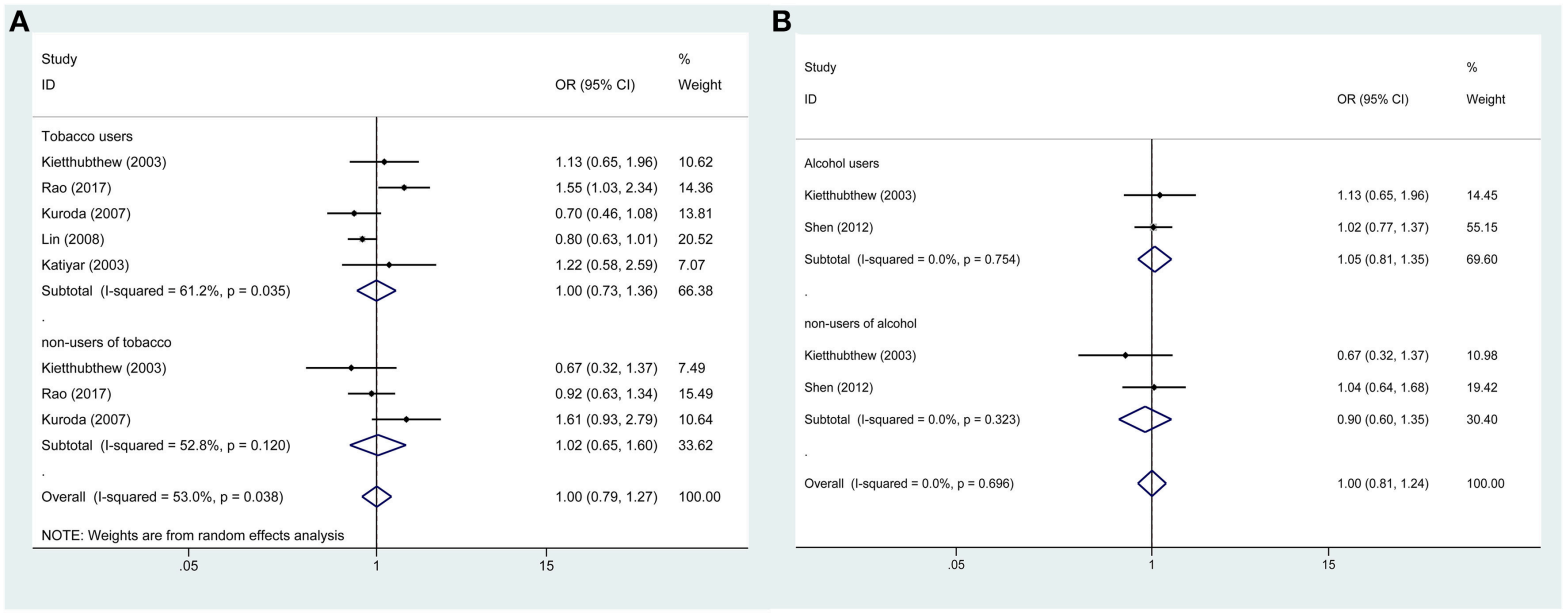
Forest plots demonstrated the association between TP53 codon 72 polymorphism and oral carcinoma susceptibility in the allele model. **(A)** Subgroup analysis by tobacco users. **(B)** Subgroup analysis by alcohol users.

**Figure 4:**
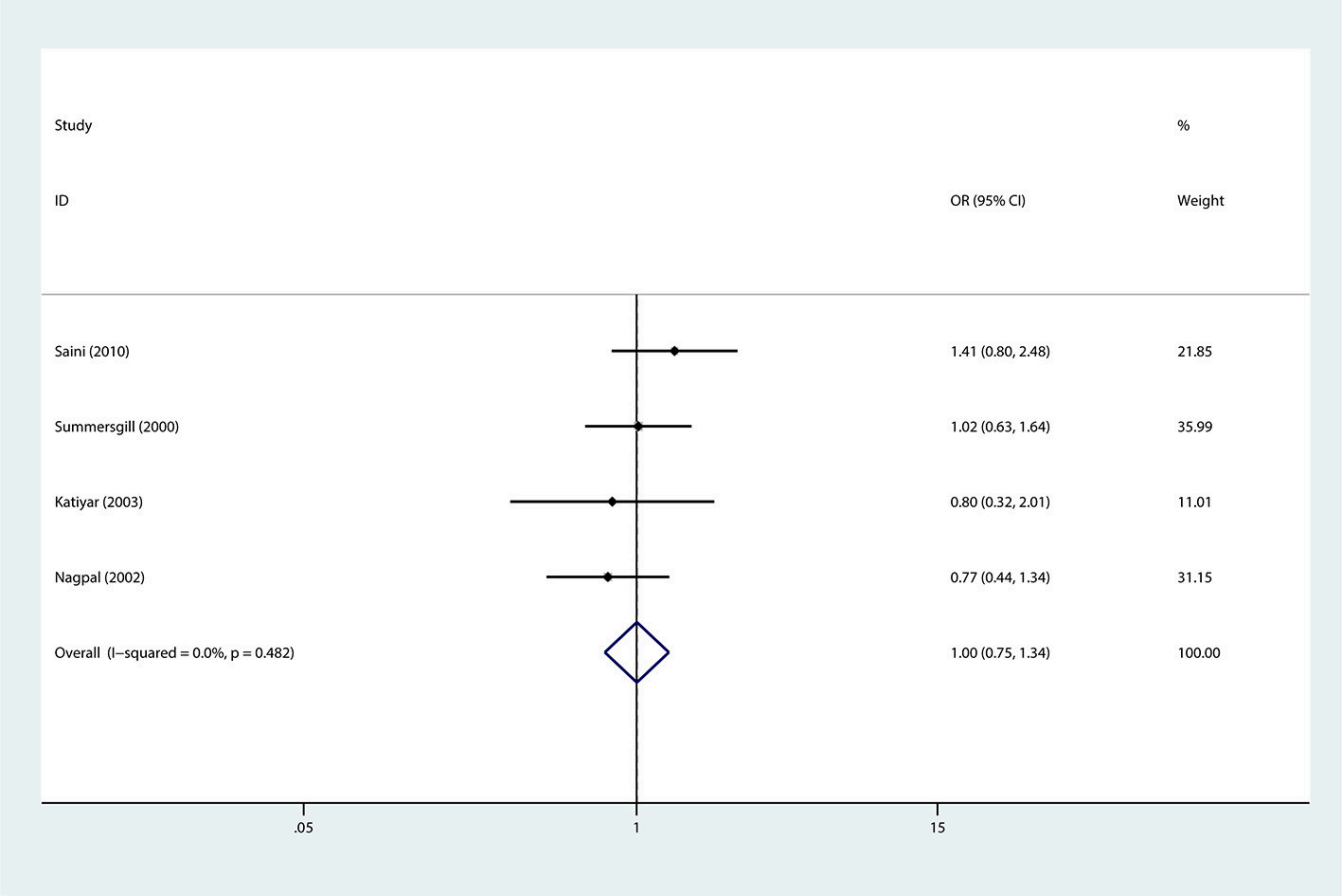
Forest plots demonstrated the association between TP53 codon 72 polymorphism and oral carcinoma susceptibility stratified by HPV infection status in the allele model.

In addition, subgroup analysis stratified by tobacco use (no vs. yes) was performed, and the association was still not significant in either tobacco users (OR = 0.88, 95%CI = 0.67–1.16) or non-users (OR = 0.84, 95%CI = 0.84–2.26). Similar results were found for subgroup analysis stratified by alcohol use or HPV infection status (Figures 3, 4, Table 3).

**Table 3:** Meta-analysis of the association between TP53 codon 72, tobacco or alcohol uses, HPV-infection status and Oral carcinoma susceptibility.

**Comparison**	**Subgroup**	**Studies**	**Heterogeneity test**		**Association test**		**Model**	**Publication bias**
			***P*-value**	***I*^2^(%)**	**OR (95%CI)**	***P*-value**		**Egger**
Pro vs. Arg	Overall	21	0	60.4	1.00 (0.90–1.12)	0.953	R	0.16
	Tobacco users	5	0.035	61.2	1.00 (0.73–1.36)	0.992	R	
	Non-users of tobacco	3	0.12	52.8	1.02 (0.65–1.60)	0.922	R	
	Alcohol users	2	0.754	0	1.05 (0.81–1.35)	0.729	F	
	Non-users of alcohol	2	0.323	0	0.90 (0.60–1.35)	0.62	F	
	HPV infection	4	0.482	0	1.00 (0.75–1.34)	0.986	F	
ArgPro vs. ArgArg	Overall	21	0.03	39	1.00 (0.90–1.11)	0.991	F	0.355
	Tobacco users	5	0.215	30.9	0.88 (0.67–1.16)	0.382	F	
	Non-users of tobacco	3	0.269	23.8	1.38 (0.84–2.26)	0.201	F	
	Alcohol users	2	0.37	0	1.13 (0.80–1.60)	0.482	F	
	Non-users of alcohol	2	0.939	0	0.93 (0.51–1.68)	0.807	F	
	HPV infection	4	0.514	0	0.90 (0.58–1.42)	0.658	F	
ProPro vs. ArgArg	Overall	21	0.001	54.4	0.97 (0.84–1.12)	0.997	R	0.399
	Tobacco users	5	0.045	58.9	1.02 (0.55–1.90)	0.953	R	
	Non-users of tobacco	3	0.227	32.7	0.99 (0.46–2.10)	0.972	R	
	Alcohol users	2	0.813	0	1.04 (0.55–1.97)	0.913	F	
	Non-users of alcohol	2	0.305	4.8	0.86 (0.38–1.91)	0.704	F	
	HPV infection	4	0.576	0	1.01 (0.55–1.85)	0.971	F	
ArgPro+ProPro vs. ArgArg	Overall	21	0.001	53.8	1.01 (0.86–1.18)	0.914	R	0.266
	Tobacco users	7	0.139	38	0.87 (0.70–1.08)	0.196	F	
	Non-users of tobacco	5	0.201	33.1	1.00 (0.70–1.44)	0.985	F	
	Alcohol users	2	0.474	0	1.11 (0.80–1.54)	0.548	F	
	Non-users of alcohol	2	0.666	0	0.91 (0.53–1.57)	0.73	F	
	HPV infection	4	0.351	9.7	1.20 (0.87–1.64)	0.267	F	
ProPro vs. ArgArg+ArgPro	Overall	21	0.033	38.4	0.96 (0.85–1.09)	0.521	F	0.356
	Tobacco users	5	0.167	38.2	0.94 (0.70–1.27)	0.7	F	
	Non-users of tobacco	3	0.501	0	0.83 (0.51–1.33)	0.435	F	
	Alcohol users	2	0.888	0	0.92 (0.50–1.67)	0.779	F	
	Non-users of alcohol	2	0.248	24.9	0.84 (0.40–1.78)	0.657	F	
	HPV infection	4	0.237	29.2	1.09 (0.66–1.81)	0.73	F	

*OR, odds ratio; CI, confidence interval; F, fixed-effects model; R, random-effects model; NA, not available; PB, population-based; HB, hospital-based*.

### Sensitivity and heterogeneity analysis

**Figure 5:**
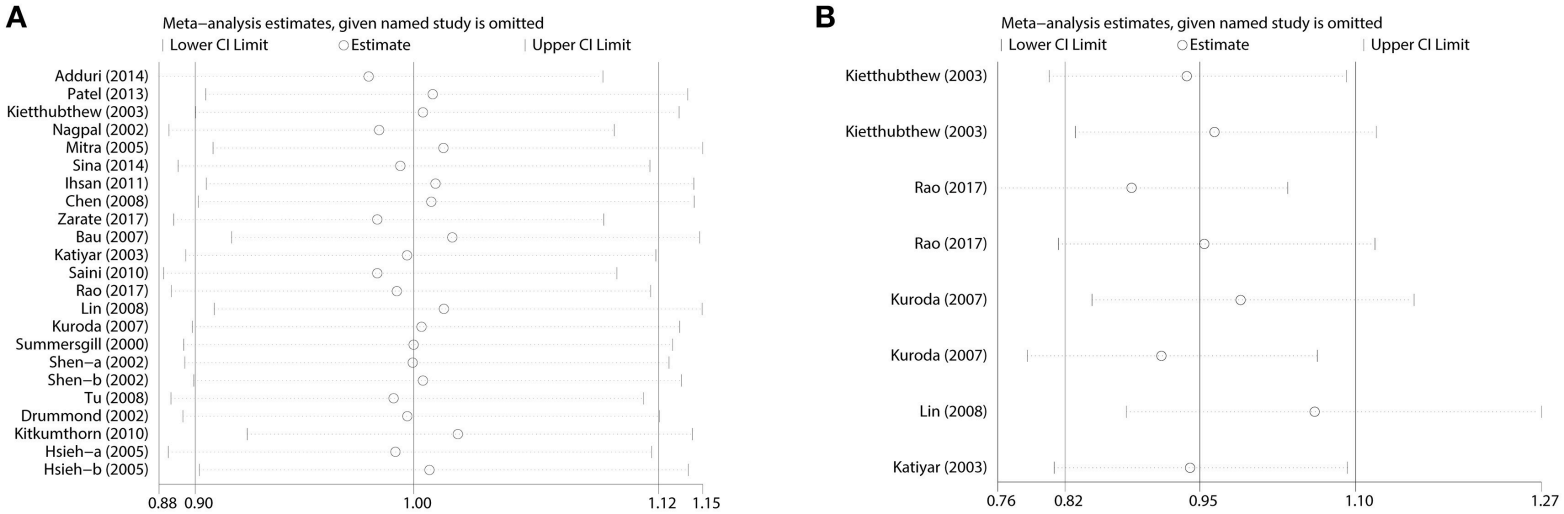
Sensitivity analysis for the influences of TP53 codon 72 polymorphism and oral carcinoma susceptibility under the allele model. **(A)** Overall analysis. **(B)** Subgroup analysis by tobacco users.

Between-study heterogeneity was examined and significant heterogeneity (*P* < 0.05) was detected in some genetic comparisons, so random-effects model was adopted (DerSimonian and Laird, 1986); otherwise, the fixed-effect model was utilized (Mantel and Haenszel, 1959). The sensitivity analysis was carried out through sequential exclusion of any one individual study, and the results showed that our conclusion was robust and credible (Figure 5).

### Publication bias

**Figure 6:**
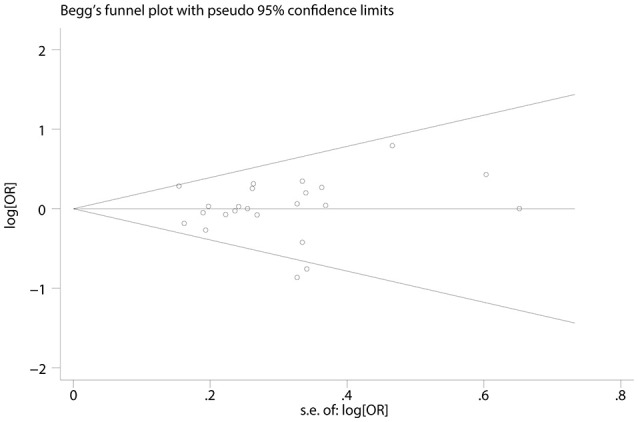
Funnel plot of publication biases on the association between TP53 codon 72 polymorphism and oral carcinoma susceptibility.

Begg's and Egger's test was utilized to examine the potential publication bias of the studies (Begg and Mazumdar, 1994; Egger et al., 1997). As shown in Figure 6, there was no significant publication bias (Tables 2, 3).

### Discussion

TP53 inactivation is a frequent event in cancer and involves point mutations and allelic loss (Baker et al., 1990; Tommasino et al., 2003). Moreover, TP53 polymorphisms could affect cancer susceptibility (Whibley et al., 2009). Pro72 allele has been implicated in coronary artery disease (Khan et al., 2016), systemic lupus erythematosus (Lee et al., 2012) and ulcerative colitis (Vaji et al., 2011). In contrast, Arg72 allele is implicated in pilocytic astrocytoma (Mascelli et al., 2016). Codon 72 TP53 polymorphisms have shown different associations with the risk of carcinomas in different populations, including oral carcinoma susceptibility (Tandle et al., 2001; Nagpal et al., 2002; Hsieh et al., 2005; Wang et al., 2012, 2014; Dahabreh et al., 2013; Saleem et al., 2013).

Although many case-control studies investigated the association of tobacco and/or alcohol uses, TP53 codon 72 polymorphism, and HPV infection with oral carcinoma susceptibility, the results were inconclusive. A past case-control studies failed to detect any significant association of TP53 codon 72 polymorphism with oral carcinoma susceptibility (Summersgill et al., 2000). In 2007, Bau et al. reported that the ArgArg genotype seemed to increase the susceptibility to oral carcinoma 2.7-fold in Chinese (Bau et al., 2007). Previous several meta-analyses reported the lack of association between TP53 codon 72 polymorphism and the risk of oral carcinoma (Zhuo et al., 2009b; Zeng et al., 2014; Hou et al., 2015), but these studies did not stratify the conditions such as tobacco and/or alcohol uses, HPV-infection status to perform subgroup analysis. Therefore, we performed the present meta-analysis to provide better estimate on the association of TP53 codon 72 polymorphism with oral carcinoma susceptibility.

Tobacco smoking is a well-known risk factor of cancer and could affect gene polymorphism in oral carcinoma (Ye et al., 2008). To evaluate the association between TP53 polymorphism and oral carcinoma susceptibility in tobacco users, we analyzed all available data extracted from the included studies, and found no significant association, indicating that TP53 codon 72 polymorphism is not a potential risk factor of oral carcinoma in tobacco users.

Regular alcohol consumption is associated with an increased risk for oral cancer. Such association is dose-dependent. Indeed, among individuals consuming 4–5 drinks daily, the risk for cancer of the oral cavity is 2–3-fold higher than among non-drinkers (Baan et al., 2007; Seitz and Stickel, 2007; Wiseman, 2008). To further investigate a possible association between oral carcinoma susceptibility and TP53 codon 72 polymorphism in alcohol users, we extracted relevant data from two studies and the results also failed to suggest a market correlation, demonstrated that TP53 codon 72 polymorphism may not be a risk of oral carcinoma in alcohol use status.

HPV infection has been suggested as one of the contributing factors for oral carcinoma. It was suggested that the interaction of TP53 codon 72 polymorphism with HPV was associated with oral carcinoma susceptibility (Kitkumthorn et al., 2010). However, this opinion was challenged by other studies (Summersgill et al., 2000; Lin et al., 2008). In this study we performed subgroup analysis on the interaction of p53 gene polymorphism with HPV infection on oral cancer susceptibility and the results indicated that TP53 codon 72 polymorphism is not a risk factor of oral carcinoma no matter HPV infection status.

The present study had several limitations. Firstly, only studies written in English or Chinese were included in the meta-analysis. This means that eligible studies published in other languages may have been overlooked, which may have introduced selection bias. Secondly, the sample size of some studies was limited and the results should be interpreted carefully. Thirdly, this study had statistical heterogeneity, although this is extremely common in meta-analyses of genetic association studies. We thus conducted subgroup analyses to identify all factors that contribute to the heterogeneity. Finally, other factors such as the age, gender, life-style that may affect the interaction of TP53 codon 72 polymorphism with oral carcinoma could not be analyzed due to the lack of original data.

In summary, this meta-analysis suggests that there is no statistical association between TP53 codon 72 polymorphism and oral cancer susceptibility, independent of tobacco and/or alcohol use and HPV-infection status. However, this conclusion should be confirmed by multi-center and large-scale studies based on multiple ethnic groups.

## Author contributions

Y-ML, JS, X-TZ, and R-HL conceived and designed the experiments. Y-ML performed the experiments. X-HY, CH, and X-WJ analyzed the data. X-WJ, Y-DY, C-JW, E-MZ, and Z-XY contributed reagents, materials, analysis tools. Y-ML wrote the paper. Y-ML, and X-TZ methods analysis.

### Conflict of interest statement

The authors declare that the research was conducted in the absence of any commercial or financial relationships that could be construed as a potential conflict of interest.
